# Prediction of Excitation Energies for Conjugated Oligomers and Polymers from Time-Dependent Density Functional Theory

**DOI:** 10.3390/ma3053430

**Published:** 2010-05-24

**Authors:** Jianmin Tao, Sergei Tretiak, Jian-Xin Zhu

**Affiliations:** 1 Theoretical Division and Center for Nonlinear Studies, Los Alamos National Laboratory, Los Alamos, NM 87545, USA; 2 Center for Integrated Nanotechnology, Los Alamos National Laboratory, Los Alamos, NM 87545, USA

**Keywords:** time-dependent density functional theory, excitation energy, optical absorption, light-emitting conjugated oligomers, **PACS** 78.67.-n, 71.15.Mb, 78.40-q, 71.45.Gm

## Abstract

With technological advances, light-emitting conjugated oligomers and polymers have become competitive candidates in the commercial market of light-emitting diodes for display and other technologies, due to the ultralow cost, light weight, and flexibility. Prediction of excitation energies of these systems plays a crucial role in the understanding of their optical properties and device design. In this review article, we discuss the calculation of excitation energies with time-dependent density functional theory, which is one of the most successful methods in the investigation of the dynamical response of molecular systems to external perturbation, owing to its high computational efficiency.

## 1. Introduction

Development of high-performance organic light-emitting diodes (OLEDs) [1,2] plays a crucial role in the fabrication of high-resolution, full-color, and flat-panel displays [3,4]. The advantage of the OLEDs over the conventional inorganic semiconductor materials such as silicon and germanium is ultralow cost, light weight, and flexibility. Furthermore, because of the ability to deposit organic films on any low-cost substrates [4,5,6,7,8] such as glass, plastic, or metal foils, OLED materials are particularly well suited for large-area displays [5]. Successful commercial production of organic electrophotographic imaging [9,10,11] for copiers, printers, and projection TV benefits from the improvement of material properties and optimization of device structure for OLEDs to enhance brightness, power efficiency, luminescence efficiency, and color purity of the three primary electroluminescence (EL) colors (red, green, and blue).

One important milestone in the development of molecular electronics is the discovery of electroluminescent conjugated oligomers and polymers [12,13,14,15]. The attraction of these materials lies at their versatility, because their physical properties such as color purity and emission efficiency can be fine-tuned by manipulation of their chemical structures. The systematic modification of the properties of emissive oligomers and polymers by synthetic design has become a vital component in the optimization of light-emitting devices [16]. Conjugated polymer has a very complicated structure. Its properties [17,18,19,20,21,22,23,24,25,26] can be affected by disorder [27] and van der Waals (vdW) interaction [28,29] that are very difficult to treat within the ground-state theories. Due to the torsional disorder effects [30,31,32], optical properties of finite chain segments can well represent those of polymers of infinite chain length. A common feature of nano-scale oligomers [33,34,35] and polymers is that they have a backbone chain with overlapping *π* orbitals. In other words, they exhibit the property of a semiconductor, because the *π* orbitals form delocalized valence and conduction bands.

Fabrication of high-resolution, full-color, and flat-panel displays [4] depends upon many factors. Apart from the optimization of device structure for OLEDs, a crucial step to improve the device performance is to design and synthesize new materials with improved properties [9,10,11,36,37,38] in charge conductivity, electroluminescence efficiency and power efficiency, thermal stability, operational lifetime, brightness, and color purity. Ideal organic EL materials [8] should be (i) readily processible, (ii) thermally stable (to withstand inevitable Joule heating generated during OLED operation), and (iii) simultaneously have high charge conductivity [36,37,38] and high luminescence efficiency. However, design and synthesis of such light-emitting organic materials with satisfactory multifunctional properties for high-performance OLEDs remain challenging.

Theoretical understanding of their optical absorption or electronic excitations is significantly important in computer-aided design and optimization of the electroluminescent oligomers and polymers. Calculation of this property poses a major challenge to both computational physicists and chemists. The difficulty lies in the fact that, in the study of the ground-state properties, one can rely on a variational principle, which enables powerful tools such as quantum Monte Carlo methods [39] and coupled cluster methods [40], but in time-dependent situations, the absence of a practical variational principle has significantly hindered the development of equally powerful methods. Several methods have been proposed to simulate the optical absorption. For example, the GW approximation [41] first proposed by Hedin [42] has been widely used to calculate the excitation energy of bulk insulators and semiconductors [43]. A much cheaper but still accurate way to calculate this quantity is time-dependent density functional theory (TDDFT) [44]. With the advent of reliable density functionals, it has become the most popular method in the study of excitation energies for finite systems and are gaining popularity for solids as well.

However, most of the calculations have been performed on small molecules. Recently we have tested [45] a sophiscated nonempirical density functional and its one-parameter hybrid version on small molecules and found that the calculated excitation energies are in fairly good agreement with experiment. Motivated by their practical success on small molecules, we then applied [46,47] them and several other commonly-used density functionals to the study of the optical absorption spectra of light-emitting conjugated oligomers. A striking difference between small molecules and conjugated oligomers is that electronic excitations of this kind of oligomers are often accompanied with some amount of charge transfer, and even singlet-triplet excitation can occur [48] (see discussion in section V). To further explore the capability of the TDDFT approach, we have applied [49] these adiabatic TDDFT methods to the calculation of excitation energies of polymers or oligomers of a large number of repeating units. Interesting enough, we have found that the accuracy of calculated excitation energies, whether arising from dominant singlet-singlet transition or from singlet-triplet transition, largely depends upon the torsional angles found from the ground-sate DFT geometry optimization. Then an interesting question arises regarding how the number of repeating units affects the torsional angles. To answer this question, we will perform more calculations on these conjugated polymers or oligomers in future.

This article is organized as follows. In the next section, we will give a brief review of this approach. For a more detailed description, see Refs.[50,51]. In section III, we discuss the performance of the TDDFT adiabatic density functionals in the calculation of the excitation energies based on the recent test on atoms and small molecules. In section IV, we discuss the application of TDDFT to light-emitting conjugated oligomers. Then further application of these density functionals with the adiabatic TDDFT formalism is discussed in section V. Finally concluding remarks are made in section VI.

## 2. Time-dependent Density Functional Theory

### 2.1. TDDFT linear response theory

TDDFT is the most important extension of Kohn-Sham ground-state DFT. It follows the Kohn-Sham strategy and maps the complicated problem of interacting electrons to a simpler problem of non-interacting electrons in an effective time-dependent potential $v_{s}\left(r,t\right)$ that yields the density n(r,t) of the interacting system. The motion of the non-interacting electrons satisfies the time-dependent Kohn-Sham single-particle equation:

(1) $$\begin{array}{c} \left[-\frac{1}{2}\nabla^{2}+v_{s}\left(r,t\right)\right]\psi_{i}\left(r,t\right)=i\frac{\partial }{\partial t}\psi_{i}\left(r,t\right), \end{array}$$

(2) $$\begin{array}{c} v_{s}=v\left(r,t\right)+\int d^{3}r^{\prime }\frac{n(r^{\prime },t)}{|r-r^{\prime }|}+v_{\text{XC}}\left(r,t\right). \end{array}$$

The instantaneous density can be calculated from the Kohn-Sham orbitals $\psi_{i}\left(r,t\right)$ by

(3) $$\begin{array}{c} n\left(r,t\right)=\sum_{i}^{\text{occ}}{\left|\psi_{i}\left(r,t\right)\right|}^{2}. \end{array}$$

Since Equations (1) and (3) are coupled via Equation (2), equation (1) must be solved self-consistently. In Equation (2), $v_{\text{XC}}\left(r,t\right)$ is the time-dependent exchange-correlation (XC) potential defined by $v_{\text{XC}}\left(r,t\right)\equiv \delta A_{\text{XC}}\left[n\right]/\delta n\left(r,t\right)$, with $A_{\text{XC}}\left[n\right]$ being the time-dependent XC functional or XC action, the analogue of the static functional $E_{\text{XC}}\left[n_{0}\right]$. It includes all unknown many-body effects. In the linear response, the density and the effective potential can be written as the sum of a large ground-state component and a small time-dependent part, *i.e.*, $n\left(r,t\right)=n_{0}\left(r\right)+n_{1}\left(r,t\right)$ and $v_{s}\left(r,t\right)=v_{s,0}\left(r\right)+v_{s,1}\left(r,t\right)$, where the effective ground-state potential $v_{s,0}\left(r\right)$ is the sum of three contributions, the external potential, the Hartree potential, and the XC potential of the ground state, *i.e.*, $v_{s,0}\left(r\right)=v_{0}\left(r\right)+u_{H,0}\left(r\right)+v_{\text{XC},0}\left(r\right)$. The effective perturbation $v_{s,1}\left(r,t\right)$ is given by $v_{s,1}\left(r,t\right)=v_{1}\left(r,t\right)+u_{H,1}\left(r,t\right)+v_{\text{XC},1}\left(r,t\right)$.

Physical excitation energies can be calculated as poles of the true linear response function, but not as poles of the single-particle Kohn-Sham response function. However, while the latter can be easily obtained from the Kohn-Sham orbitals (see below), the former must be calculated from the complicated correlated wave function whose exact form remains unknown. Within the TDDFT formalism, the physical excitation is calculated as the sum of the Kohn-Sham excitation energy and a small energy shift due to the many-body effects, from the linear response theory [52,53] via the density-density response function [50] $\chi (r,r^{\prime },t,t^{\prime })$, in which the only unknown part is the XC kernel defined by

(4) $$\begin{array}{c} f_{\text{XC}}\left(r,r^{\prime },t,t^{\prime }\right)\equiv \delta v_{\text{XC}}\left(\left[n\right];r,t\right)/\delta n\left(r^{\prime },t^{\prime }\right). \end{array}$$

The key idea is that the exact linear density response of an interacting system to the external perturbation is equivalent to the linear density response of a noninteracting system to the effective perturbation, i.e,

(5) $$\begin{array}{ccc} n_{1}\left(r,\omega \right) & = & \int dr\;\chi \left(r,r^{\prime },\omega \right)v_{1}\left(r^{\prime },\omega \right)\\ & = & \int dr\;\chi_{s}\left(r,r^{\prime },\omega \right)v_{s,1}\left(r^{\prime },\omega \right)\;, \end{array}$$

where

(6) $$\begin{array}{ccc} v_{s,1}\left(r,t\right) & = & v_{1}\left(r,t\right)+\int dr^{\prime }\frac{n_{1}\left(r^{\prime },t\right)}{|r-r^{\prime }|}\\ & + & \int_{-\infty }^{t}dt^{\prime }\int dr^{\prime }f_{\text{XC}}\left(r,r^{\prime },t,t^{\prime }\right)n_{1}\left(r^{\prime },t\right), \end{array}$$

and $\chi_{s}\left(r,r^{\prime },\omega \right)$ is the Kohn-Sham response function evaluated with the Kohn-Sham ground-state orbitals. For spin-unpolarized systems, we have

(7) $$\begin{array}{c} \chi_{s}\left(r,r^{\prime };\omega \right)=2\sum_{j,k}\left(n_{k}-n_{j}\right)\frac{\phi_{k}^{*}\left(r\right)\phi_{j}\left(r\right)\phi_{j}^{*}\left(r^{\prime }\right)\phi_{k}\left(r^{\prime }\right)}{\omega -\omega_{jk}+i\delta }, \end{array}$$

where $n_{k}$ are the orbital electron occupation numbers. By substituting the effective perturbation $v_{s,1}\left(r,t\right)$ into Equation (5) with the observation that the poles $\omega_{jk}$ of the Kohn-Sham response function are generally different from those of the interacting system, one arrives at an equation [53], from which excitation energies of the interacting system can be calculated as an eigenvalue problem. The detailed discussion for the calculation of the excitation energies within the TDDFT linear response theory have been documented in Refs. [50,52,53,54]. After computing the excitation energies, the corresponding optical transition strengths are obtained from the transition dipole moments calculated as expectation values of the dipole operator using the respective transition amplitude. Transition dipole moments and excitation energies constitute the essential ingredients for modeling optical spectra.

### 2.2. Adiabatic approximation for the time-dependent XC potential

In TDDFT, everything is known, except for the time-dependent dynamical XC potential or kernel (see Equation (4) for definition), which has to be approximated in practice. The simplest construction of the dynamical XC potential is the adiabatic approximation [55], which takes the form of the ground-state XC potential but replaces the ground-state density $n_{0}\left(r\right)$ with the instantaneous density n(r,t), namely,

(8) $$\begin{array}{c} v_{\text{XC}}^{\text{ad}}\left(\left[n\right];r,t\right)=\frac{\delta E_{\text{XC}}\left[n_{0}\right]}{\delta n_{0}\left(r\right)}{|}_{n_{0}\left(r\right)=n\left(r,t\right)}\;. \end{array}$$

Within the adiabatic approximation the XC kernel can be calculated from

(9) $$\begin{array}{c} f_{\text{XC}}^{\text{ad}}\left(r,r^{\prime },t,t^{\prime }\right)\equiv \frac{\delta v_{\text{XC}}\left(\left[n_{0}\right];r\right)}{\delta n(r^{\prime })}\delta \left(t-t^{\prime }\right), \end{array}$$

which is local in time, while it is not necessarily local in space. Since it ignores the frequency dependence arising from the XC vector potential [56,57,58,59] (*i.e.*, it forgets the history prior to *t*) and thus retardation and dissipation effects [60,61,62], this (time-frozen) approximation fails to describe multi-particle excitations [63,64] or charge transfer states [65,66,67]. Nevertheless, it is a good approximation if the system evolves slowly in time or if the nonequilibrium state is not too far from equilibrium. Moreover, it has been shown [53,68] that (at least for small systems) the largest source of error in the prediction of low-lying excitation energies arises from the approximation for the ground-state XC potential. This justifies the adiabatic approximation in the calculation of low-lying excitations of atoms and molecules. Due to the simplicity in both theoretical construction and numerical implementation, the adiabatic approximation has been widely used to calculate low-lying single-particle excitation energies [54,69,70,71,72,73,74,75,76]. The low-lying excited states in the visible and near-UV region (namely near-ultraviolet region that ranges from 400nm down to 200 nm) are the most interesting ones. For example, photodissociation often proceeds on the lowest excited potential energy surface, and the photoemmision wavelength of materials is controlled by the lowest electronic excitations. A quantitative description of electronic excited states of molecules is important in spectroscopy, photochemistry, and the design of optical materials (e.g., design of dyes). The detail of the TDDFT linear response theory for the calculation of the excitation energies within the adiabatic approximation has been documented in the literature [52,53,69].

To date a ladder of sophiscated density functionals $E_{\text{XC}}\left[n_{0}\right]$ have been proposed [77,78]. The first three rungs of the ladder of nonempirical density functionals are formed, respectively, by the local spin density approximation (LSDA) which only uses the electron densities $n_{\sigma }$ as its local ingredients, the generalized gradient approximation (GGA) of Perdew, Burke, and Ernzerhof (PBE) [79] which employs not only the electron densities but also density gradients $\nabla n_{\sigma }$, and the meta-GGA of Tao, Perdew, Staroverov, and Scuseria (TPSS) [80,81] which makes use of the Kohn-Sham kinetic energy densities defined by

(10) $$\begin{array}{c} \tau_{\sigma }\left(r\right)=\sum_{k=1}^{\text{occup}}\frac{\hbar^{2}}{2m}{\left|\nabla \psi_{k\sigma }\left(r\right)\right|}^{2}\;\left(\tau \left(r\right)=\sum_{\sigma }\tau_{\sigma }\left(r\right)\right), \end{array}$$

as the additional local ingredients, where the $\psi_{k\sigma }$ are the occupied Kohn-Sham orbitals. Here “nonempirical” means that a density functional does not contain any parameter fitted to experiment, because all the parameters introduced in the assumed functional form are determined by the exact constraints imposed. According to the adiabatic connection [82,83], the performance of pure density functionals can be improved by mixing into small amount of exact exchange [84]. The cost for this improvement is the slight increase of computational time. Because hybrid functionals are orbital-dependent, their potential must be evaluated via the chain rule of functional derivative [85]. Motivated by the argument based on the adiabatic connection, hybrid functionals based on pure density functionals have been proposed. For example, the popular functional PBE0 [86,87] is a one-parameter hybrid density functional which is constructed from the nonempirical PBE GGA,

(11) $$\begin{array}{c} E_{\text{XC}}^{\text{PBE}0}=aE_{x}^{\text{exact}}+\left(1-a\right)E_{X}^{\text{PBE}}+E_{C}^{\text{PBE}}, \end{array}$$

with *a* = 2.5 being the exact-exchange mixing coefficient. The widely-used functional B3LYP [88] is a three-parameter hybrid density functional with 20% exact exchange mixed in its exchange component. With the introduction of exact exchange, a hybrid functional, however, does not satisfy any universal constraint beyond those satisfied by its parent pure density functional, while it improves the description of the asymptotic behavior of the pure density functional potential. Nevertheless, this improvement turns out to be helpful in most cases [89].

Finally we point out that, in general, a non-adiabatic correction to the adiabatic approximation is needed even in the low-frequency limit. Non-adiabatic corrections for both homogeneous and inhomogeneous systems within the linear response regime have been proposed [56,59,60]. Recently a quite promising approach, which is called “quantum continuum mechanics” [90,91], in analogy with classical theories of continuous media (elasticity and hydrodynamics), has been developed to describe the dynamics of quantum many-body systems without explicit reference to the individual particles of which the system is constituted. Although these higher-level approximations are quite complicated from both theoretical and computational points of view, they have shed light on the treatment of difficult problems such as multi-particle excitations, charger transfer, vdW interaction, etc. Calculations of the excitation energies beyond the adiabatic approximation can be found in Refs. [92,93,94,95]. In this article, we only focus on the adiabatic TDDFT excitation energies.

## 3. Excitation Energies of Atoms and Small Molecules

It has been shown [54,69,70,71,72] that the adiabatic TDDFT yields the excitation energies of molecules with fairly good accuracy. We tested [45] the capability of the adiabatic TPSS meta-GGA [80,81] and its one-parameter hybrid version TPSSh [89] (a hybrid of TPSS with 10% exact exchange) to describe low-lying excitations for eleven atoms with *Z* ≤ 36 (He, Li, Be, Ne, Na, Mg, Ar, K, Ca, Zn, and Kr) and prototype small molecules CO, N_2_, H_2_O, CH_2_O (formaldehyde), (CH_3_)_2_CO (acetone), C_2_H_4_ (ethylene), C_6_H_6_ (benzene), and C_5_H_5_N (pyridine). Since the TPSS meta-GGA is constructed from the PBE GGA [79], and PBE GGA is constructed from the LSDA, the LSDA and PBE GGA were also included in this test. The results are compared to both experiment and those obtained with two popular hybrid functionals PBE0 and B3LYP.

All calculations were performed using the GAUSSIAN 03 suite [96]. Vertical excitation energies of molecules were calculated using the self-consistent ground-state geometries optimized with respective density functionals. A relatively large basis set 6-311++G(3df,3pd) was used in all the calculations of atoms and small molecules. The mean error (m.e.) (or signed error) was calculated using the sign convention: error = theory - experiment.

### 3.1. Atoms

Table 1 shows two lowest-lying singlet excitation energies of the selected atoms. They were calculated with the adiabatic LSDA, PBE GGA, PBE0, B3LYP, TPSS meta-GGA, and TPSSh functionals. Experimental values [97] are also listed for comparison.

From Table 1 we observe that all six adiabatic density functionals produce remarkably accurate excitation energies, with mean absolute error (m.a.e.) of 0.5 eV. From the mean errors (m.e.), we can see that all nonhybrid functionals (LSDA, PBE GGA, and TPSS meta-GGA) slightly underestimate low-lying excitation energies of atoms, while all hybrid functionals (PBE0, B3LYP, and TPSSh) yield overestimates. The error with mixed sign suggests the difficulty of further systematic improvement from the nonadiabatic corrections [56,59]. This is resonant with the finding made from time-dependent current-density functional theory [95].

### 3.2. Small molecules

Theoretical prediction or interpretation of discrete molecular electronic excitation spectrum is of significant importance. Many physical and chemical properties of materials are directly related to electronic excitations. We calculated [45] low-lying excitation energies of our test set, which includes three inorganic (CO, N_2_, H_2_O) and five organic (CH_2_O, (CH_3_)_2_CO, C_2_H_4_, benzene, pyridine) molecules. The results are reported in Tables II–IX, respectively.

Table 2, Table 3 and Table 4 display the vertical excitation energies of three prototype inorganic molecules CO, N_2_, and H_2_O. For the CO molecule, as shown in Table II, the adiabatic TPSS functional produces the vertical (low-lying) excitation energies in better agreement with the experimental values [98] than the adiabatic PBE GGA, while it is slightly less accurate than the adiabatic LSDA. As expected, the adiabatic TPSSh yields further improvement over the TPSS meta-GGA. Mixing small amount of the exact exchange into a semilocal functional improves the asymptotic behavior of the XC potential and the description of nodal regions, both of which a pure density functional has difficulty to treat. Similar results are observed for the N_2_ molecule, an iso-electron series of the CO molecule. As observed in Table IV, both TPSS and TPSSh functionals describe the vertical excitations of water molecule well and produce the low-lying excitation energies more accurately than the adiabatic LSDA and PBE GGA. As expected, the best results are obtained with the adiabatic hybrid functionals PBE0, B3LYP, and TPSSh. We can see from the mean errors in Tables II–IV that all the density functionals tend to underestimate the molecular excitation energies.

Table 5, Table 6, Table 7, Table 8 and Table 9 show the excitation energies of five organic molecules formaldehyde, acetone, ethylene, benzene, and pyridine. The TPSS meta-GGA consistently provides a more realistic description of the excitation energies of molecules than the PBE GGA, and shows an overall improvement over LSDA. TPSSh gives further improvement upon the TPSS functional, and achieves a comparable accuracy of PBE0 and B3LYP. As observed in Table 2, Table 3 and Table 4, these density functionals tend to underestimate the excitation energies of molecules.

Table 10 shows the mean absolute relative errors of these functionals. We can see from Table 10 that the overall order of accuracy for these functionals is

(12) $$\begin{array}{c} \text{PBE}<\text{LSDA}≲\text{TPSS}<\text{TPSSh}≲B3\text{LYP}<\text{PBE}0. \end{array}$$

The mean absolute relative error of each density functional tested here is less than 10%, suggesting the good performance of the TPSS and TPSSh functionals for the description of atomic and molecular excitations. The systematic underestimate of the excitation energies of molecules within the TDDFT-adiabatic approximation suggests that further improvement can be made by going beyond the adiabatic approximation.

In summary, the test of the TDDFT-adiabatic TPSS meta-GGA and TPSSh hybrid functionals on atoms and molecules show that both density functionals produce the vertical excitation energies in fairly good agreement with experiment and improve upon the LSDA and PBE GGA. This suggests that both TPSS and TPSSh functionals within the adiabatic approximation are capable of describing photochemically interesting phenomena when the system is exposed to a time-dependent laser field. Compared to other nonhybrid density functionals, TPSS yields the best performance, while TPSSh can achieve the comparable accuracy of the most popular hybrid functionals B3LYP and PBE0. In view of the good performance of the TPSS functional for diverse systems and a wide class of properties, we conclude that TPSS is indeed a reliable nonhybrid universal functional, which can serve as a platform from which higher-level approximations can be constructed [106].

## 4. Absorption Spectra of Blue-Light Emitting Oligoquinolines

From section III, we see that, like other commonly-used density functionals, TPSS and TPSSh functionals perform well in the calculation of excitation energies of small molecules within the TDDFT adiabatic approximation. In order to make a comprehensive assesment of the density functionals that were originally developed for ground-state properties, we applied [46,47] these functionals to complex systems [30,71,76,107]. In our assesment of the performance of several popular density functionals for conjugated oligomers and polymers that will be discussed in section V, the PBE GGA functional has been excluded, because it gives the least accurate excitation energies of atoms and small molecules in comparison with other density functionals we tested. Our choice of model systems is based on the following considerations: (i) there should be similarities and differences in structure between small molecules we tested above and the model systems employed in the next test, (ii) relevant experimental measurements of high quality are available so that we can make a comparison between theory and experiment, and (iii) the systems should be of potential use or have been employed as nanomaterials in the commercial market. Consequently we selected a family of *n-type* (electron transport) light-emitting conjugated oligomers as our model systems.

**Figure 1:**
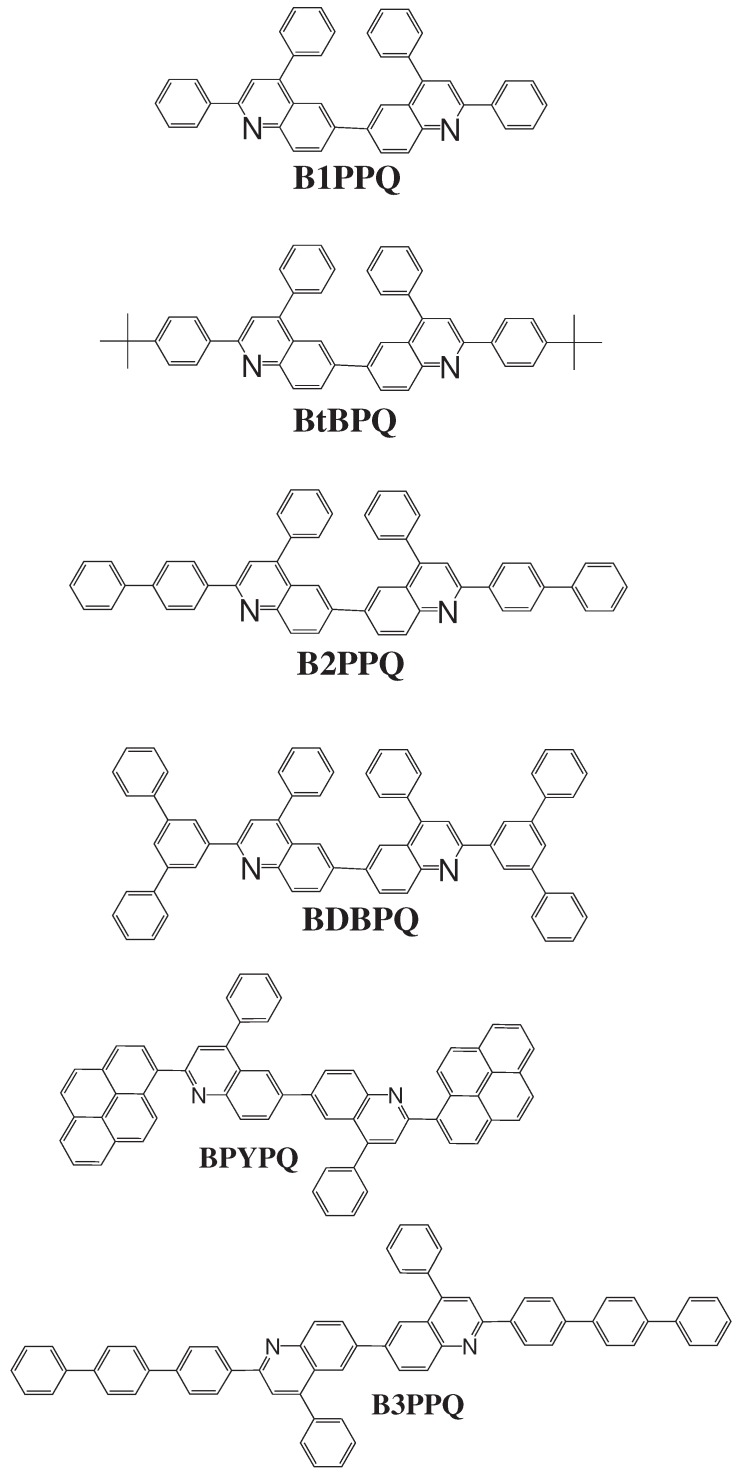
Molecular structures of the computationally studied blue-light-emitting oligoquinolines.

In recent years, these organic materials have been increasingly gaining popularity in the development of OLEDs. In particular, Jenekhe and collaborators [1,108] have synthesized a series of *n-type* blue-light-emitting *π*-conjugated oligomers [109] (see Figure 1). They found that these organic materials can be used to fabricate high-efficiency light-emitting diodes. These oligoquinolines, 6,6-bis(2,4-diphenylquinoline) (B1PPQ), 6,6-bis(2-(4-tert-butylphenyl)-4-phenylquinoline) (BtBPQ), 6,6-bis(2-p-biphenyl)-4-phenylquinoline)(B2PPQ), 6,6-bis((3,5-diphenylbenzene)-4-phenylquinoline) (BDBPQ), 6,6-bis(2-(1-pyrenyl)-4-phenylquinoline) (BPYPQ), and 6,6-bis(2-(1-triphenyl)-4-phenylquinoline) (B3PPQ) (See Figure 1 for molecular structures) exhibit many desirable properties of organic materials for developing high-performance light-emitting diodes: good blue color purity, high brightness, high efficiency, and high glass-transition temperatures. In particular, the two pyrenyl- and triphenyl-bearing oligoquinoline molecules BPYPQ and B3PPQ have many desirable properties such as excellent thermal stability, high melt transitions, high quantum yields, and bright blue electroluminescence with high efficiency, and are highly emissive electron transport materials for OLEDs and have been used as emitters in recent fabrication of OLED devices. The optical properties of these light-emitting oligomers such as absorption and emission spectra have been experimentally measured as well [1,108].

Conjugated oligomer is typically a finite segment of polymer chain with several repeating units. Its characteristic absorption spectrum is in the UV/visible region. When the number of repeating monomeric units reaches some number, it mimics well the corresponding polymer it constitutes [110,111]. Study of the absorption spectra of oligomers can help us to better understand the optical properties of polymers.

To provide a deep physical insight into these phenomena, we calculated [46,47] the optical absorption of oligoquinolines in gas phase and chloroform (CHCl_3_) solution, respectively, with the adiabatic TDDFT methods. The excitation energies of oligoquinolines in solution were calculated with PCM (polarizable continuum model) [112]. Our calculations show that the first peak of optical absorption corresponds to the lowest singlet excited state, whereas several excited states that are degenerate or nearly-degenerate, contribute to the experimentally observed higher-frequency peak. We find that the lowest excitation energies of oligoquinolines in chloroform (CHCl_3_) solution calculated with the adiabatic hybrid functional PBE0 are in good agreement with experiment. We also calculated the oscillator strengths and dipole moments of the oligoquinoline molecules both in gas phase as well as in chloroform solution. We see that both oscillator strength and dipole moment are larger in solution than in gas phase, as expected. These two quantities are directly related to the peak magnitude or absorption intensity in the UV/visible absorption spectra. By comparing the simulated absorption spectra in gas phase with those in chloroform solution, we find that, relative to the excitation energy in gas phase, there is a consistent redshift in excitation energy in solution, due to the solute-solvent interaction.

**Figure 2:**
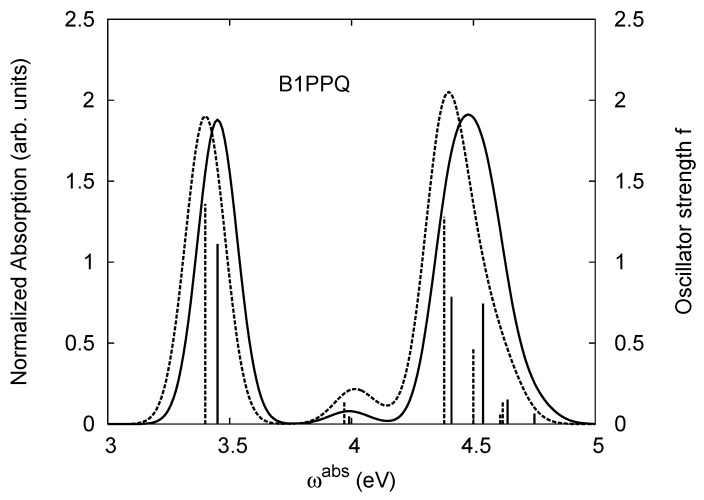
Normalized absorption *I* of Equation (13) (in arbitrary units) (right side) and oscillator strength *f* (left side) of B1PPQ. The solid and dashed curves represent the normalized absorption in gas phase and solution, while the solid and dashed “sticks” represent the oscillator strength in gas phase and solution, respectively. The absorption wavelength $\lambda^{\text{abs}}$ (in units of nm) may be obtained from the relation $\lambda^{\text{abs}}=\left(1239.84\mathrm{eV}/\omega^{\text{abs}}\right)\left(\mathrm{nm}\right)$, where $\omega^{\text{abs}}$ is the absorption frequency (in units of eV).

**Figure 3:**
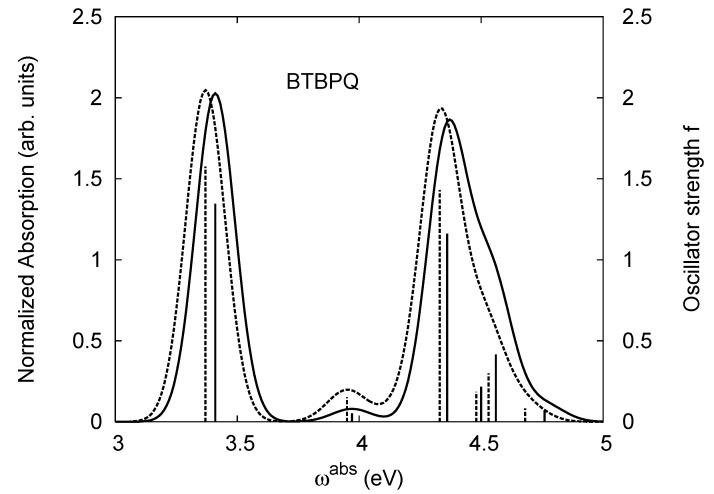
The same as Figure 2, but for BtBPQ.

**Figure 4:**
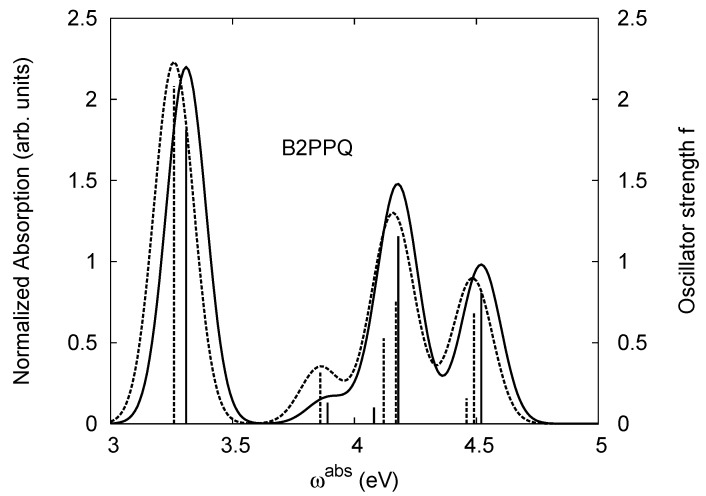
The same as Figure 2, but for B2PPQ.

**Figure 5:**
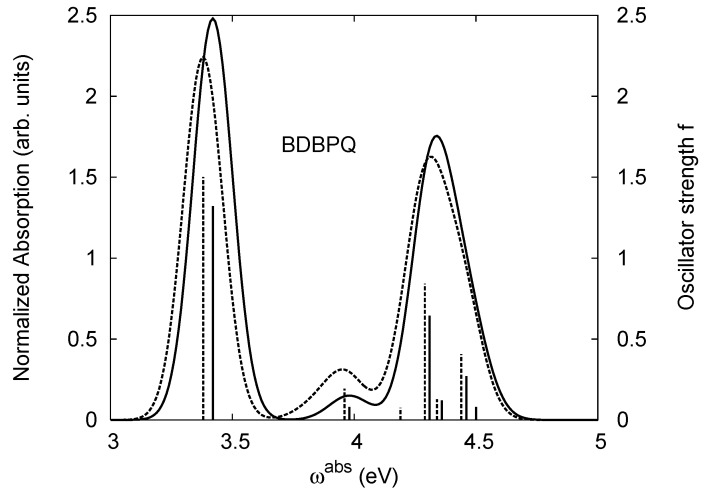
The same as Figure 2, but for BDBPQ.

Table 11, Table 12, Table 13, Table 14, Table 15 and Table 16 show the summary of selected TDDFT excited-state quantities of oligoquinolines (B1PPQ, BtBPQ, B2PPQ, BDBPQ, BPYPQ, B3PPQ, see Figure 1) in gas phase and solution, respectively. To simulate the experimentally observed absorption with the calculated data (see Figure 2, Figure 3, Figure 4 and Figure 5), we assume that the normalized absorption intensity or peak magnitude takes the analytic expression of

(13) $$\begin{array}{c} I\left(\omega \right)=\sum_{i}f\left(\omega_{i}\right)\delta_{m}\left(\omega -\omega_{i}\right)/\sum_{i}f\left(\omega_{i}\right), \end{array}$$

where $\delta_{m}\left(x\right)$ is a *δ*-like function defined by

(14) $$\begin{array}{c} \delta_{m}\left(x\right)=\frac{m}{\pi }\frac{1}{1+m^{2}x^{2}}, \end{array}$$

with the properties [113] of $\int_{-\infty }^{\infty }dx\;\delta_{m}\left(x\right)=1$ and $\lim_{m\to \infty }\delta_{m}\left(x\right)\to \delta \left(x\right)$. Here *m* is determined by a fit to experiment. The fitted values are m = 15.5 for the B1PPQ, BtBPQ, B2PPQ, and BDBPQ oligomers in both gas phase and solution, m = 5.0 for BPYPQ, and m = 7.0 for B3PPQ. The calculated absorption spectra are plotted in Figure 2, Figure 3, Figure 4 and Figure 5. In our simulation, we did not employ the most commonly-used gaussian function. These two functions [113] (Equation (14) and gaussian function) have similar properties and are equivalent in the limit of *m* → ∞, but the former gives a better fit to experiment.

These excited states were studied using the natural transition orbital analysis [114] based on the calculated transition density matrices. This analysis offers the most compact representation of a given transition density in terms of an expansion into single-particle transitions. As representative examples, the calculated transition orbitals of B1PPQ and BtBPQ are displayed in Table 17 and Table 18. For other oligomers, see Refs. [46,47].

### 4.1. B1PPQ and BtBPQ

The TDDFT energies of the lowest excited state of B1PPQ in gas phase listed in Table 11 show a pronounced blueshift along the density functional models used from LSDA to meta-GGA to hybrid functionals. We observe a strong sensitivity to the fraction of the exact orbital exchange used in the functional. The total blueshift when going from LSDA to PBE0 (functional with 25% portion of the exact orbital exchange) is about 0.8 eV. Due to nearly non-polar structure of the molecule, we observe a fairly small solvatochromic shift [115] of about 50 meV. The calculated PBE0 value for the excitation energy in chloroform, 3.40 eV, agrees well with the experimental maximum (3.48 eV) of the lowest absorption peak. We note that such comparisons can be done only approximately, since vibrational progression and disorder effects are not considered in the present calculations. Such phenomena can account up to 0.1 ∼ 0.2 eV difference [116].

The lowest excited state of B1PPQ has a sizable oscillator strength. Due to Kasha’s rule [117], this state is also responsible for molecular luminescence, where the oscillator strength defines an efficiency of this process. The calculated oscillator strength tends to increase with the increase of the fraction of the exact orbital exchange in the functional. We observe about 50% difference between computed LSDA and PBE0 values of the oscillator strength. In contrast to the energy values, solvent leads to a noticeable increase of the oscillator strength, compared to the gas phase values. Trends observed for calculated ground state dipole moment values (see Table 11), are very similar to those for oscillator strengths. (The latter are directly relevant to the respective transition dipole moments from the ground state to excited state).

Experimental optical absorption [1] of B1PPQ has a second peak appearing at 4.43 eV. Our calculations consistently predict that two excited states with nearly the same oscillator strength contribute to the intensity of this peak. These states are separated by about 0.1 eV across all density functionals used. However, similar to the lowest state, the blueshift up to 1 eV is observed when going along the line of density functionals employed, from LSDA to PBE0. These two higher-lying states have very similar solvatochromatic shifts as well. Again, PBE0 provides the most accurate transition energy values when compared to the experimental data. It is interesting to note that the oscillator strength of these two higher-lying states grows dramatically in the hybrid functionals. We observe 4-6 fold increase when going from LSDA to PBE0. According to the LSDA (TPSS) results, the second peak magnitude should be much smaller compared to that of the first one. This is not, however, the case of experiment, where the second peak has larger amplitude compared to the first one [1]. PBE0 nearly captures the experimental observations. Figure 2 displays the oscillator strength of B1PPQ as a function of absorption frequency *ω* (solid “stick” in gas phase and dashed “stick” in solution) and our simulation of Equation (13) for the normalized absorption band intensity as a function of *ω* (solid curve in gas phase and dashed curve in solution) obtained from the PBE0 results. We note that the second peak has more intensity (integrated area under the curve), since it is composed from the two overlapping electronic transitions.

For reference, Table 11 lists calculated energies of the first triplet state. Triplet states are important for efficiency of the light-emitting devices based on organic conjugated molecules. Their energetics and delocalization properties affect the dynamics of the charge recombination [118,119]. Moreover, the lowest triplet state is responsible for weak phosphorescence in such systems [120,121] . We note that the calculated energies of the first triplet state does not change substantially for all methods, which means that the singlet/triplet gap splitting grows significantly from nonhybrid to hybrid functionals, reaching 1.2 eV for PBE0 model. Such theoretical prediction is likely close to the experimental case, since such organic molecules generally exhibit large values of singlet/triplet gap due to low dimensionality and quantum confinement [120].

To analyze the electronic nature of calculated singlet excited states we utilized a natural transition orbital representation, as shown in Table 17 and Table 18 for B1PPQ and BtBPQ. (See Refs. [46,47] for B2PPQ, BPYPQ, and B3PPQ.) We plotted the orbitals derived from PBE0 computational results, since this method provides the most accurate results in comparison with experiment across the entire molecular family considered. We first note that all considered excited states are *π*-π* excitations, as illustrated by their transition orbitals. The lowest excited state |1〉 can essentially be represented by a single-pair of transition orbitals (see Table 17). This is a delocalized excitation involving the conjugated backbone of the B1PPQ oligomer. The side phenyl rings do not participate substantially in this optical excitation. Excited states |8〉 and |9〉 contributing to the second absorption peak are mainly delocalized in the middle section of the molecule. We notice that state |8〉 is multiconfigurational, *i.e.*, it can be represented by several dominating pairs of transition orbitals. Here only the dominant contribution is shown in Table 17.

BtBPQ has an identical conjugated molecular structure with B1PPQ. Methylated *σ*-bonded ends at BtBPQ do not introduce any substantial effects into excited-state electronic structure. Nevertheless, these methyls yield consistent small redshifts of the first lowest (and dominant) singlet-singlet excitation energies of BtBPQ (in order of several tenth of meV) across all computational results and experimental data compared to those of B1PPQ (see Table 11 and Table 12). A slightly more noticeable effect is an increase of the oscillator strength of the lowest excited state, |1〉, in BtBPQ. This can be rationalized by examining the respective transition orbitals (see Ref. [46]) where the elongated molecular ends provide slightly larger room for electronic delocalization, which is reflected in the values of the dipole moments. Compared to B1PPQ, the intensity in the higher energy absorption peak is shifted toward the lower state. Notably, both solvent effect and methylation (BtBPQ) lead to this effect (compare Figure 2 and Figure 3). Finally, we emphasize that slightly different chemical structure of B1PPQ and BtBPQ has no effect on the energies of their first triplet states.

### 4.2. B2PPQ and BDBPQ

Compared to B1PPQ, molecular structure of B2PPQ has longer conjugated backbone, whereas BDBPQ features four aryl substituents at meta-positions at both ends (see Figure 1). Even though the main physical phenomena and trends for B1PPQ discussed above are the same for B2PPQ and BDBPQ, here we emphasize a few observed differences due to different molecular compositions.

The energy of the lowest singlet molecular state of B2PPQ is redshifted compared to that in both B1PPQ and BtBPQ. This is a direct consequence of elongation of the conjugation length [122]. This shift is not very significant in calculations and less pronounced in experiment due to torsional distortion between aryls at the ends which disrupts conjugation. Nevertheless, the terminal aryls are well participating in this excitation (see Table 18). In contrast, the aryl substitutions in BDBPQ do not have any substantial effect on the lowest-state excitation energy, which is closest to that in BtBPQ, compared to the other five members of the family. It is well established that the electronic delocalization through the meta-position in the phenyl ring substitutions is effectively blocked [123,124,125] and such substitutions do not usually bear significant effects. Consequently, the electronic state does not delocalize on the four terminal phenyls, as illustrated by the respective transition orbitals (see Ref. [46] for detail). In fact, relative ordering of the energy of the first excited state observed in experiment [1] (from blue to red, B1PPQ, BDBPQ, BtBPQ, B2PPQ, B3PPQ, BPYPQ) is well reproduced by all computational methods (Table 11, Table 12, Table 13 and Table 14). Compared to B1PPQ, we further observe that, due to the extended conjugation, the value of the oscillator strength increases substantially in B2BPQ. This effect is smaller in BDBPQ.

The energetics of the two higher-lying excited states contributing to the second absorption peak is substantially changed due to aryl substitutions in B2PPQ and BDBPQ. Noteworthy, these two excitations are even more multi-configurational. For example, excited state |13〉 in B2PPQ is a mixture of two transitions between pairs of transition orbitals: the first pair corresponds to the transition in the middle section of the molecules, involving the “middle” aryls, whereas the second pair describes charge transfer from the terminal phenyls to the center (see Table 18). Roughly a similar picture holds for excited state |10〉 in BDBPQ (see Ref. [46] for detail). Such high energy excited states can be delocalized through the barrier imposed by meta-positions on the molecular structure. Indeed both contributions to excited state |13〉 in BDBPQ represent partial charge re-distribution from the terminal phenyls to the center of the oligomer.

Spectroscopically, we observe splitting of the second peak in the simulated absorption spectrum of B2PPQ (see Figure 4), where both maxima have smaller magnitudes, compared to the first absorption peak. Experimentally, only one absorption peak is observed in the higher-frequency region in B2PPQ (not shown). However, its intensity is substantially lower, compared to the other molecules in the family. Among other computed properties, the energies of the lowest triplet state are the same for three molecules (B1PPQ, BtBPQ, and BDBPQ), and show only a moderate redshift for B2PPQ, BPYPQ, and B3PPQ, due to extended conjugation length (see Table 11, Table 12, Table 13, Table 14, Table 15 and Table 16). Triplet states also display minimal solvatochromic shifts (about 10 meV) and typically have very localized nature [75]. Note that the ground state dipole moment (which is approximately directed orthogonally to the molecular backbone) is roughly the same in B1PPQ, BtBPQ, BDBPQ, and B2PPQ oligomers and slightly larger in BDYPQ and B3PPQ.

### 4.3. BPYPQ and B3PPQ

From Table 15 we observe that the first or lowest-frequency peak of BPYPQ occurs at 3.00 eV (the experimental value is 3.26 eV) with the largest oscillator strength *f* = 1.82, and a higher-frequency peak occurs at 3.60 eV (the same as the experimental value) with the oscillator strength *f* = 0.48, almost three times smaller than the largest oscillator strength. The third or the highest-frequency absorption peak occurs at about 4.09 eV (4.34 eV for the experimental measurement), with even a smaller oscillator strength *f* = 0.1. The oscillator strength of the third absorption peak is underestimated significantly with the B3LYP and PBE0 functionals. The LSDA, TPSS, and TPSSh functionals yield a more realistic oscillator strength, although it is still too small, compared to the experimental observation, where the experimental intensity of the third absorption band is quite noticeable [108]. This discrepancy of theory from experiment for the third peak absorbance may arise from many effects such as temperature, disorder, vibrational progression, etc. These factors have not been taken into consideration in our calculations. We also observe a persistent redshift for the first two peaks from gas phase to solution. This redshift (of about 10 meV) also occurs for the lowest triplet excitation. The dipole moment of BPYPQ is vanishingly small if all the atoms are in a same plane, due to its high symmetry. However, this geometry is not the ground-state geometry. In the ground state, there are dihedral angles between two benzene rings connected by a *σ*-bond. These dihedral angles effectively reduce the symmetry of the molecule, resulting in a large ground-state dipole moment. Compared to the dipole moments of B1PPQ, BtBPQ, B2PPQ, and BDBPQ, the dipole moments of BPYPQ and B3PPQ is larger, due to their relatively larger size. While the effect of the solvent-solute interaction on the optical absorption is small, it has a significant effect on the ground-state dipole moment and causes the noticeable increase of the oscillator strength in solution, compared to that in gas phase. The absorptions calculated with other adiabatic TDDFT functionals are in fairly good agreement with experiment. The accuracy increases when we go from LSDA, TPSS, TPSSh, B3LYP, to PBE0.

Table 16 shows that in gas phase the first two absorptions of B3PPQ occur at 3.27 eV and 4.02 eV, respectively, with the oscillator strength of the first peak being about twice that of the second peak. Interestingly, our calculation shows that there should be another absorption peak, which occurs at a higher frequency 4.30 eV. The absorption intensity of the third peak is nearly the same as the second. Since these two peaks are located closely, they may combine to form a broader single peak. Therefore, we may only observe two absorption peaks in total in the experiment. In solution, the three peaks are expected to occur at slightly lower frequency, due to the redshift, as shown in Table 16. The solvent-solute effects on the absorption and the ground-state moment are the same as those for BPYPQ.

The natural transition orbital analysis for excited states of BPYPQ and B3PPQ showns that B3PPQ orbitals are slightly less delocalized, compared to those of BPYPQ, while the molecular structure of the former has a longer backbone. This is reflected by the higher excitation energies of B3PPQ. The same trend for the lowest triplet excitation is also observed by comparing Table 15 with Table 16. From the natural transition orbital analysis, we can also see that these selected excited states arise from *π*-π* excitations. See Ref. [47] for detailed discussion.

## 5. Excitation Energies of Conjugated Polymers

Application of TDDFT to complex systems or conjugated oligomers discussed in section III shows that the adiabatic TDDFT density functionals we investigated here continue to yield excitation energies in good agreement with experiment. Encouraged by this, we further applied these TDDFT methods to study a more complicated class of systems–conjugated polymers, which is closely connected with conjugated oligomers [126]. However, in the simulation of electronic excitations of small molecules and oligomers, the effort has been devoted to the study of the absorption arising from singlet-singlet excitation, leaving the singlet-triplet excitation less investigated [127,128]. An important reason for this omission is that triplet-state energies are not easy to measure through direct optical absorption due to very low singlet-triplet (*S*_0_ − *T*_1_) absorption coefficient [129] and low phosphorescence quantum yield [121] (< 10^−6^). The major approaches to probe triplet states in conjugated polymers are the charge recombination energy transfer, and singlet-triplet (*T*_1_ − *S*_0_ or *S*_1_ − *T*_1_) intersystem crossing [130,131,132].

It has been found [120,133] that the properties of the triplet states directly impact device performance, as discussed in section IV.A. Therefore, investigation of triplet excitations is crucial for a full understanding of electroluminescence behavior of conjugated polymers and for the improvement of new materials. Monkman and collaborators [120] investigated the photophysics of triplet states in a series of conjugated polymers, poly(3-octylthiophene) (P3OT), poly(2-butyloxy-5-octylphenyl-3-thiophene) (PBOPT), poly(2-methoxy-5-(2’-ethylhexyoxy)-p-phenylenevinylene) (MEHPPV), poly(dioctylflourene) (PFO), poly(2,5-hexyloxyphenylenevinylene) (DHOPPV), poly(2,5-pyridinediyl) (PPY), poly(2-methoxy-5-(2’-ethylhexyoxy)-p-phenylenecyanovinylene) (CN-MEHPPV), and polyemeraldine (PANi), and measured the excitation energies of the lowest singlet- and triplet-excited states. Their measurements show that the excitation energies in general respect the well-known rule of thumb found for small molecules:

(15) $$\begin{array}{c} E_{T}\approx 2E_{S}/3, \end{array}$$

where *E_T_* is the triplet excitation energy and *E_S_* is the singlet-singlet excitation energy.

**Figure 6:**
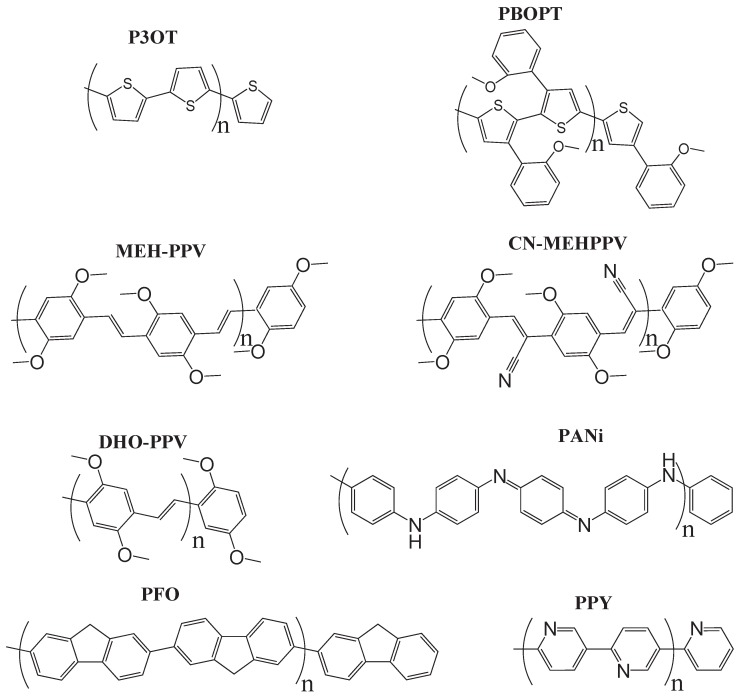
Chemical structures of the computationally studied light-emitting congugated polymers.

Usually a polymer has very long chain length. In practical calculations, we can choose several repeating monomeric units, because at some critical length, optical properties of finite chain segments well represent those of polymers of an infinite chain. Moreover, due to disorder, *infinite chains* of polymers are thought to be finite segments [122,134,135,136]. The polymers we studied have chain length of ∼10 nm (See Figure 6 for chemical structures). The segment of this chain length contains at least 16 molecular rings, which mimics quite welll the optical properties of polymers with infinite chain [110,111]. The groups of -(CH_2_)_*n*_CH_3_ has little effect on the optical properties of the polymers [46,47]. These side chains only affect some physical and chemical properties, such as phase transition temperature, solubilities, etc. and thus can be removed from the backbone of polymers in calculations.

Numerical calculations [49] show that the accuracy of the calculated TDDFT excitation energies largely depends upon the dihedral angles obtained by the ground-state DFT geometry optimization. When the DFT torsional dihedral angles are close to experimental estimates, the TDDFT excitation energies agree well with experiment. This trend is observed based on calculations of eight different polymeric systems considered here. It is shown that, while hybrid density functionals can respect the thumb rule of Equation (15), nonhybrid functionals do not, suggesting inadequacy of semilocal functionals in predicting the triplet excitation energies for polymers.

Table 19 shows the first singlet and triplet excitation energies of the polymers in gas phase calculated with the adiabatic TDDFT. The experimental results are also listed for comparison. The number of “molecular” rings included in our calculations for each polymer is given in the parentheses in Table 19 and Table 21. These numbers are chosen so that the lengths of the polymers are about 10 nm. This size effect [137] will be reduced by increasing the repeating units. However, adding the repeating units will simultaneously increase the computational time. On the other hand, high accuracy usually can be achieved by using large basis set, which will result in significant increase in computational time. In practical calculations, we can use 6-31G basis set, which is relatively smaller than those used in small molecular calculations, and we should prepare the polymers with moderate length of chain. This is a balanced choice between the size effect and the accuracy we can tolerate.

From Table 19 we observe that, among the five adiabatic TDDFT methods, the adiabatic PBE0 functional yields the most accurate excitation energies. This is consistent with our previous studies [46,47]. We can see from Table 19 that the difference between the singlet and the triplet excitation energies, *E_S_* − *E_T_*, is ∼ 0 − 0.1 eV for LSDA, ∼ 0 − 0.2 eV for TPSS, ∼ 0.5 eV for TPSSh, ∼ 0.6 eV for B3LYP, and ∼ 0.8 eV for PBE0. The difference increases as the amount of exact exchange increases. However, some studies suggest [110,138] that for semilocal density functionals (LSDA, GGA, and meta-GGA), this difference may vanish in the limit of infinite chain length, a result similar to the performance of semilocal functionals for solids. Mixing exact exchange into a semilocal functional will (i) partly correct the errors from self interaction, (ii) improve the asymptotic behavior of the XC potential, (iii) improve the description of nodel regions of the Kohn-Sham orbitals, and (iv) build in other many-body properties such as excitonic effects [110,138] which have not been taken into account properly in pure density functional approximations and thus will lead to a finite difference in this limit.

Interestingly, we find that, when the theoretical dihedral angle is smaller than the experimental dihedral angle, the TDDFT methods tend to underestimate the excitation energies regardless of whether the excitation is singlet or triplet. When the theoretical dihedral angle is close to the experimental one, the TDDFT excitation energies are in good agreement with experiment. Our calculations show that, in rare cases, theoretical dihedral angles can be greater that experimental estimates. In this case, the excitation energies are overestimated by the TDDFT methods. A comparison of the dihedral angles between DFT and experimental or other accurate theoretical estimates is displayed in Table 20. The origin of torsional angles (or generally tortional disorder) of polymers is complicated. It may arise from interchain interaction in amorphous polymeric materials [30,31,139,140] or from the vdW interaction [29] between phenyl rings, which have not been taken into consideration in current DFT methods.

The excitation energies of the polymers in benzene solvent are summarized in Table 21. From Table 21, we can see that the lowest singlet-singlet excitation energies in solution have a redshift of ∼ 0.01 − 0.05 eV, compared to those in gas phase (Table 19). This solvent stabilization is attributed to a strong *S*_0_ − *S*_1_ transition dipole moment and is consistent with what we have observed for oligomers [46,47]. However, this trend does not apply to the triplet excitation which has no dipolar strength. Triplet excitation energies are nearly the same whether the polymer is in gas phase or in solution.

Finally we point out that the validity of the trend “TDDFT excitation energies are in good agreement with experiment only when the theoretical torsions agree with experimental estimates” we have found is based on our calculations of eight polymers. This trend may not be automatically valid for other polymeric systems. Our calculations show that a semilocal functional without exact exchange mixing does not satisfy the well-known “two-third” thumb rule relation between the singlet-singlet and singlet-triplet excitation energies. For semilocal functionals, the difference in energy between singlet state and triplet state is less than 0.1 eV for polymers with chain length of 10 nm and may vanish in the limit of infinite chain length. Compared to semilocal functionals, hybrid functionals yield much larger difference between singlet-singlet and singlet-triplet excitation energies for polymers with finite chain length. This difference increases with more exact exchange mixed in semilocal functionals, and is nonzero even in the limit of infinite chain length.

## 6. Conclusion

In conclusion, we have discussed the calculation of excitation energies with the TDDFT approach and the test on atoms and small molecules. Then we have reviewed our recent applications of TDDFT to the simulation of the optical absorptions and excitations of conjugated oligomers and polymers. The calculations are carried out with several widely-used density functionals and the results are compared with experiments. We find that the adiabatic TDDFT methods constructed from the ground-state density functionals yield excitation energies in good agreement with experiment for small molecules and conjugated oligomers. For polymers we tested, the accuracy of calculated excitation energies is largely determined by the torsional angles calculated from the ground-state DFT geometry optimization. Only if the calculated torsional angles are close to experiment, can high accuracy of TDDFT excitation energies be achieved. Our calculation also shows that conjugated oligomers and polymers often exhibit large singlet/triplet gap, which may arise from low dimensionality and quantum confinement [120,145].

The origin of the torsional angles is complicated. It may be related to the disorder effect or the interchain vdW interaction or both. To estimate the vdW effect, we can employ a vdW-corrected TDDFT, which can be constructed from the ground-state DFT [29], to these complex systems. Another interesting question we will look at is how important the number of repeating units on the torsional angles. We will also apply this approach to other conjugated polymers/oligomers to see whether the torsional angles have the same effect on the excitation energies of the polymers/oligomers we discussed here.

Finally we conclude our review by pointing out that the order of accuracy of the five adiabatic density functionals given by Equation (12) in the prediction of the low-lying excitation energies of small molecules [45] continue to hold for large systems such as conjugated oligomers and polymers. Since conjugated oligomers of finite chain segment are a bridge between small molecules and conjugated polymers of infinite chain length, study of conjugated oligomers, the subject of this special issue, is of general interest.

## Figures and Tables

**Table 1:** Two lowest-lying singlet excitation energies (in eV) of atoms calculated using six functionals with the basis set 6-311++G(3df,3pd). The mean error (m.e.) (with the sign convention that error = theory - experiment) and the mean absolute error (m.a.e.) are also shown. The mean experimental value of these atoms is 8.06 eV. (1 hartree = 27.21 eV).

Atom	Transition	LSDA	PBE	TPSS	TPSSh	PBE0	B3LYP	Expt^*a*^
He	1*s* → 2*s*	19.59	19.73	20.27	20.58	20.62	20.50	20.62
	1*s* → 2*s*	22.99	23.41	24.04	24.23	24.05	23.95	21.22
Li	2*s* → 2*p*	1.98	1.98	1.99	1.97	1.95	1.98	1.85
	2*s* → 3*s*	3.12	3.09	3.09	3.13	3.23	3.16	3.37
Be	2*s* → 2*p*	4.84	4.91	5.06	5.05	4.94	4.88	5.28
	2*s* → 3*s*	6.11	6.12	6.29	6.35	6.32	6.21	6.78
Ne	2*p* → 3*s*	17.45	17.21	17.55	17.94	18.27	17.88	16.62
	2*p* → 3*p*	19.82	19.46	19.74	20.16	20.59	20.11	18.38
Na	3*s* → 3*p*	2.25	2.12	2.02	2.02	2.08	2.23	2.10
	3*s* → 4*s*	3.05	2.91	2.87	2.90	3.02	3.02	3.19
Mg	3*s* → 3*p*	4.24	4.18	4.18	4.19	4.20	4.23	4.35
	3*s* → 4*s*	5.02	4.93	5.01	5.06	5.08	5.00	5.39
Ar	3*p* → 4*s*	11.32	11.27	11.59	11.81	11.90	11.56	11.55
	3*p* → 4*p*	12.68	12.50	12.74	13.00	13.22	12.89	12.91
K	4*s* → 4*p*	1.70	1.50	1.36	1.36	1.45	1.64	1.61
	4*s* → 5*s*	2.52	2.35	2.28	2.30	2.42	2.43	2.61
Ca	4*s* → 3*d*	1.88	1.88	1.87	2.02	2.24	2.16	2.71
	4*s* → 4*p*	3.09	2.98	2.90	2.90	2.96	3.03	2.93
Zn	4*s* → 4*p*	5.80	5.67	5.59	5.52	5.51	5.65	5.80
	2*s* → 5*s*	6.38	6.12	6.10	6.12	6.20	6.22	6.92
Kr	4*p* → 5*s*	9.52	9.43	9.72	9.92	10.01	9.69	9.92
	4*p* → 5*s*	10.84	10.64	10.85	11.10	11.30	10.98	11.30
m.e.		-0.06	-0.14	-0.02	0.12	0.19	0.09	…
m.a.e.		0.47	0.51	0.49	0.50	0.50	0.47	…

^*a*^From Ref. [97].

**Table 2:** Low-lying excitation energies (in eV) of CO calculated using six functionals with the basis set 6-311++G(3df,3pd). Calculations are performed using the geometry optimized on respective functionals with the same basis. The mean error (m.e) and the mean absolute error (m.a.e.) are also shown. The mean experimental value is 9.58 eV.

Symmetry	LSDA	PBE	TPSS	TPSSh	PBE0	B3LYP	Expt^*a*^
^3^Π	5.98	5.68	5.75	5.78	5.77	5.89	6.32
^3^Σ^+^	8.45	7.97	7.88	7.88	7.96	8.03	8.51
^1^Π	8.19	8.19	8.40	8.50	8.49	8.47	8.51
^3^Δ	9.21	8.59	8.53	8.59	8.70	8.71	9.36
^3^Σ^−^	9.90	9.31	9.64	9.92	9.89	9.80	9.88
^1^Σ^−^	9.94	9.79	10.05	10.15	9.89	9.86	9.88
^1^Δ	9.90	9.72	9.96	10.01	10.29	10.26	10.23
^3^Σ^+^	9.55	9.72	9.96	10.01	10.05	9.92	10.40
^3^Σ^+^	10.48	10.21	10.60	10.86	10.94	10.85	11.30
^1^Σ^+^	10.73	10.62	10.89	11.15	11.31	11.32	11.40
					
m.e.	-0.35	-0.60	-0.41	-0.30	-0.25	-0.28	…
m.a.e.	0.36	0.60	0.45	0.36	0.27	0.28	…

^*a*^ From Ref. [98].

**Table 3:** The same as Table 2, but for N_2_. The mean experimental value is 9.38 eV.

Symmetry	LSDA	PBE	TPSS	TPSSh	PBE0	B3LYP	Expt^*a*^
Σu+3	7.96	7.42	7.22	7.12	7.14	7.25	7.75
^3^Π_g_	7.62	7.34	7.43	7.54	7.64	7.68	8.04
^3^Δ_u_	8.90	8.19	8.05	8.01	8.06	8.12	8.88
^1^Π_g_	9.11	9.04	9.23	9.37	9.43	9.37	9.31
Σu−3	9.73	9.58	9.82	9.79	9.53	9.47	9.67
Σu−1	8.73	9.58	9.82	9.79	9.53	9.47	9.92
^1^Δ_u_	10.28	9.98	9.95	9.98	10.05	10.86	10.27
^3^Π_u_	10.39	10.37	10.65	10.79	10.79	10.68	11.19
					
m.e.	-0.29	-0.44	-0.36	-0.33	-0.36	-0.27	…
m.a.e.	0.36	0.44	0.40	0.38	0.39	0.43	…

^*a*^From Ref. [99].

**Table 4:** The same as Table 2, but for H_2_O. The mean experimental value is 8.99 eV.

Symmetry	LSDA	PBE	TPSS	TPSSh	PBE0	B3LYP	Expt^*a*^
^3^B_1_	6.30	6.06	6.30	6.59	6.80	6.56	7.14
^1^B_1_	6.60	6.44	6.65	6.96	7.24	6.96	7.49
^3^A_2_	7.99	7.72	7.90	8.24	8.57	8.31	9.1
^1^A_2_	8.08	7.88	8.05	8.39	8.77	8.47	9.2
^3^A_1_	8.26	8.10	8.36	8.64	8.84	8.58	9.35
^1^A_1_	8.67	8.62	8.86	9.15	9.43	9.10	9.73
^3^B_2_	9.94	9.75	9.95	10.26	10.55	10.28	9.93
^1^B_2_	10.14	10.04	10.23	10.57	10.93	10.59	10.0
					
m.e.	-0.75	-0.92	-0.71	-0.39	-0.10	-0.39	…
m.a.e.	0.78	0.93	0.77	0.62	0.49	0.62	…

^*a*^From Ref. [100,101].

**Table 5:** The same as Table 2, but for formaldehyde (H_2_CO). The mean experimental value is 6.90 eV.

Symmetry	LSDA	PBE	TPSS	TPSSh	PBE0	B3LYP	Expt^*a*^
^3^A_2_	3.15	3.09	3.26	3.30	3.22	3.26	3.5
^1^A_2_	3.75	3.82	4.06	4.12	4.02	3.99	4.1
^3^A_1_	6.37	5.75	5.57	5.46	5.43	5.58	6.0
^3^B_2_	5.89	5.68	5.95	6.27	6.53	6.38	7.09
^1^B_2_	5.99	5.89	6.11	6.45	6.77	6.53	7.13
^3^B_2_	7.10	6.91	7.17	7.44	7.62	7.46	7.92
^1^B_2_	7.18	7.07	7.29	7.58	7.82	7.61	7.98
^3^A_1_	6.86	6.63	6.87	7.21	7.50	7.35	8.11
^1^A_1_	6.95	6.82	7.01	7.36	7.72	7.47	8.14
^1^B_1_	8.86	8.82	9.01	9.15	9.22	9.09	9.0
					
m.e.	-0.69	-0.87	-0.69	-0.49	-0.31	-0.43	…
m.a.e.	0.77	0.87	0.69	0.52	0.36	0.44	…

^*a*^From Refs. [102,103].

**Table 6:** The same as Table 2, but for acetone ((CH_3_)_2_CO). The mean experimental value is 6.17 eV.

Symmetry	LSDA	PBE	TPSS	TPSSh	PBE0	B3LYP	Expt^*a*^
^3^A_2_	3.70	3.59	3.69	3.73	3.81	3.81	4.18
^1^A_2_	4.22	4.21	4.37	4.41	4.49	4.44	4.43
^3^A_1_	6.13	5.70	5.97	5.96	5.60	5.70	5.88
^3^A_2_	6.28	6.11	6.27	6.26	6.01	5.75	6.26
^1^B_2_	5.09	5.00	5.22	5.22	6.08	5.80	6.36
^1^A_2_	6.30	6.14	6.30	6.30	7.18	6.92	7.36
^1^A_1_	6.08	5.92	6.08	6.08	7.02	6.72	7.41
^1^B_1_	6.51	6.36	6.53	6.52	7.37	7.12	7.49
					
m.e.	-0.63	-0.79	-0.62	-0.61	-0.23	-0.39	…
m.a.e.	0.70	0.79	0.64	0.53	0.24	0.39	…

^*a*^From Ref. [71].

**Table 7:** The same as Table 2, but for ethylene (C_2_H_4_). The mean experimental value is 7.40 eV.

Symmetry	LSDA	PBE	TPSS	TPSSh	PBE0	B3LYP	Expt^*a*^
^3^B_1u_	4.81	4.26	4.12	4.02	3.97	4.17	4.36
^3^B_3u_	6.75	6.45	6.58	6.74	6.86	6.65	6.98
^1^B_3u_	6.82	6.58	6.67	6.84	7.01	6.75	7.15
^1^B_1u_	7.58	7.44	7.53	7.59	7.61	7.48	7.66
^3^B_1g_	6.95	6.99	7.17	7.34	7.39	7.27	7.79
^3^B_2g_	7.34	7.02	7.12	7.31	7.52	7.26	7.79
^1^B_1g_	7.36	7.16	7.25	7.43	7.60	7.34	7.83
^1^B_2g_	7.41	7.13	7.21	7.40	7.64	7.34	8.0
^3^A_g_	8.39	8.03	8.20	8.33	8.37	8.25	8.15
^1^A_g_	8.71	8.48	8.56	8.70	8.85	8.63	8.29
					
m.e.	-0.22	-0.47	-0.37	-0.25	-0.12	-0.29	…
m.a.e.	0.41	0.50	0.42	0.35	0.27	0.37	…

^*a*^From Ref. [102].

**Table 8:** The same as Table 2, but for benzene (C_6_H_6_). The mean experimental value is 5.89 eV.

Symmetry	LSDA	PBE	TPSS	TPSSh	PBE0	B3LYP	Expt^*a*^
^3^B_1u_	4.47	3.98	3.84	3.73	3.68	3.84	3.94
^3^E_1u_	4.82	4.61	4.67	4.70	4.75	4.72	4.76
^1^B_2u_	5.33	5.22	5.32	5.42	5.52	5.41	4.90
^3^B_2u_	5.05	4.89	4.98	5.06	5.12	5.07	5.60
^1^B_1u_	6.07	5.94	6.00	6.09	6.18	6.05	6.20
^1^E_1g_	6.12	5.89	5.99	6.18	6.38	6.11	6.33
^3^E_1g_	6.09	5.84	5.95	6.14	6.32	6.07	6.34
^1^A_2u_	6.70	6.43	6.50	6.69	6.90	6.62	6.93
^1^E_2u_	6.71	6.44	6.50	6.70	6.95	6.65	6.95
^3^E_1u_	6.66	6.37	6.45	6.63	6.82	6.57	6.98
					
m.e.	-0.09	-0.33	-0.27	-0.16	-0.03	-0.18	…
m.a.e.	0.30	0.40	0.36	0.26	0.17	0.28	…

^*a*^From Ref. [104].

**Table 9:** The same as Table 2, but for pyridine (C_5_H_5_N). The mean experimental value is 5.07 eV.

Symmetry	LSDA	PBE	TPSS	TPSSh	PBE0	B3LYP	Expt^*a*^
^3^B_1_	3.69	3.68	3.84	3.99	3.81	3.97	4.1
^3^A_1_	4.59	4.11	3.97	3.86	4.08	4.05	4.1
^1^B_1_	4.22	4.33	4.55	4.74	4.86	4.76	4.59
^3^B_2_	4.62	4.41	4.44	4.49	4.54	4.52	4.84
^3^A_1_	5.04	4.78	4.81	4.86	4.92	4.88	4.84
^1^B_2_	5.46	5.33	5.41	5.53	5.63	5.52	4.99
^3^A_2_	4.19	4.30	4.57	4.83	5.03	4.93	5.40
^1^A_2_	4.29	4.43	4.71	4.99	5.20	5.07	5.43
^3^B_2_	5.45	5.40	5.65	6.06	5.72	5.64	6.02*
^1^A_1_	6.03	5.97	6.18	6.31	6.41	6.23	6.38
					
m.e.	-0.31	-0.40	-0.26	-0.08	-0.05	-0.11	…
m.a.e.	0.54	0.47	0.34	0.25	0.25	0.26	…

^*a*^From Ref. [102]; *CASPT2 estimate from Refs. [71,105].

**Table 10:** Mean absolute relative error (m.a.r.e.) of the atoms and molecules listed in Table 1, Table 2, Table 3, Table 4, Table 5, Table 6, Table 7, Table 8 and Table 9.

	LSDA	PBE	TPSS	TPSSh	PBE0	B3LYP
m.a.r.e. (%)	7.3	8.3	7.1	5.7	4.4	5.3

**Table 11:** B1PPQ: Singlet and triplet vertical excitation energies ($\omega_{S}^{n}$, $\omega_{T}^{n}$, *n* = the *n*-th excited state) in eV, the transition oscillator strength ($f^{\text{abs},n}$), and the dipole moment of the ground state in Debye of B1PPQ molecule in gas phase ($\mu_{g}$) and chloroform solution ($\mu_{\text{sol}}$), calculated using the five adiabatic density functionals with the basis set 6-31G(2df,p) and the geometry optimized on the respective density functionals with the same basis. (1 eV = 8065.5 cm^−1^ = 0.03675 hartree; The energy (in units of eV) of wave length *λ* (in units of nm) is *hc*/*λ* = (nm/*λ*)1239.84 eV, where *h* is Planck’s constant and *c* is the speed of light). Experimental values measured in chloroform are obtained from Ref. [1].

	gas	gas	gas	gas	gas	gas	gas	gas	sol	sol	sol	sol	sol	sol	sol	sol
	$\omega_{S}^{\text{abs},1}$	$f^{\text{abs},1}$	$\omega_{S}^{\text{abs},10}$	$f^{\text{abs},10}$	$\omega_{S}^{\text{abs},11}$	$f^{\text{abs},11}$	$\omega_{T}^{\text{abs}}$	$\mu_{g}$	$\omega_{S}^{\text{abs},1}$	$f^{\text{abs},1}$	$\omega_{S}^{\text{abs},9}$	$f^{\text{abs},9}$	$\omega_{S}^{\text{abs},12}$	$f^{\text{abs},12}$	$\omega_{T}^{\text{abs}}$	$\mu_{\text{sol}}$
LSDA	2.70	0.745	3.37	0.197	3.49	0.121	2.26	0.708	2.66	0.948	3.38	0.122	3.50	0.194	2.67	1.052
			$\omega_{S}^{\text{abs},8}$	$f^{\text{abs},8}$	$\omega_{S}^{\text{abs},10}$	$f^{\text{abs},10}$					$\omega_{S}^{\text{abs},8}$	$f^{\text{abs},8}$	$\omega_{S}^{\text{abs},10}$	$f^{\text{abs},10}$		
TPSS	2.81	0.747	3.50	0.187	3.60	0.124	2.18	0.838	2.78	0.938	3.50	0.155	3.61	0.231	2.19	1.231
			$\omega_{S}^{\text{abs},8}$	$f^{\text{abs},8}$	$\omega_{S}^{\text{abs},13}$	$f^{\text{abs},13}$					$\omega_{S}^{\text{abs},8}$	$f^{\text{abs},8}$	$\omega_{S}^{\text{abs},11}$	$f^{\text{abs},11}$		
TPSSh	3.12	0.916	3.90	0.205	4.15	0.919	2.23	0.865	3.08	1.137	3.89	0.174	4.09	1.144	2.24	1.261
			$\omega_{S}^{\text{abs},10}$	$f^{\text{abs},10}$	$\omega_{S}^{\text{abs},11}$	$f^{\text{abs},11}$					$\omega_{S}^{\text{abs},8}$	$f^{\text{abs},8}$	$\omega_{S}^{\text{abs},9}$	$f^{\text{abs},9}$		
B3LYP	3.33	1.040	4.37	0.623	4.40	0.422	2.35	0.901	3.28	1.288	4.20	0.650	4.31	0.856	2.37	1.300
			$\omega_{S}^{\text{abs},8}$	$f^{\text{abs},8}$	$\omega_{S}^{\text{abs},9}$	$f^{\text{abs},9}$					$\omega_{S}^{\text{abs},8}$	$f^{\text{abs},8}$	$\omega_{S}^{\text{abs},9}$	$f^{\text{abs},9}$		
PBE0	3.45	1.112	4.41	0.786	4.54	0.744	2.27	0.902	3.40	1.359	4.38	1.279	4.50	0.464	2.29	1.305
									$\omega_{1\mathrm{st}}^{\text{abs}}$		$\omega_{2\mathrm{nd}}^{\text{abs}}$					
Expt									3.48		4.43					

**Table 12:** The same as Table 11, but for BtBPQ. Experimental values measured in chloroform are obtained from Ref. [1].

	gas	gas	gas	gas	gas	gas	gas	gas	sol	sol	sol	sol	sol	sol	sol	sol
	$\omega_{S}^{\text{abs},1}$	$f^{\text{abs},1}$	$\omega_{S}^{\text{abs},10}$	$f^{\text{abs},10}$	$\omega_{S}^{\text{abs},11}$	$f^{\text{abs},11}$	$\omega_{T}^{\text{abs}}$	$\mu_{g}$	$\omega_{S}^{\text{abs},1}$	$f^{\text{abs},1}$	$\omega_{S}^{\text{abs},9}$	$f^{\text{abs},9}$	$\omega_{S}^{\text{abs},12}$	$f^{\text{abs},12}$	$\omega_{T}^{\text{abs}}$	$\mu_{\text{sol}}$
LSDA	2.64	0.896	3.33	0.316	3.61	0.3685	2.24	0.699	2.61	1.095	3.34	0.535	3.59	0.485	2.25	1.010
			$\omega_{S}^{\text{abs},8}$	$f^{\text{abs},8}$	$\omega_{S}^{\text{abs},15}$	$f^{\text{abs},15}$					$\omega_{S}^{\text{abs},8}$	$f^{\text{abs},8}$	$\omega_{S}^{\text{abs},13}$	$f^{\text{abs},13}$		
TPSS	2.77	0.868	3.47	0.458	3.73	0.339	2.18	0.844	2.74	1.058	3.47	0.536	3.69	0.207	2.19	1.220
			$\omega_{S}^{\text{abs},8}$	$f^{\text{abs},8}$	$\omega_{S}^{\text{abs},13}$	$f^{\text{abs},13}$					$\omega_{S}^{\text{abs},8}$	$f^{\text{abs},8}$	$\omega_{S}^{\text{abs},11}$	$f^{\text{abs},11}$		
TPSSh	3.08	1.093	3.87	0.427	4.10	0.627	2.23	0.872	3.04	1.300	3.88	0.337	4.06	0.815	2.24	1.248
			$\omega_{S}^{\text{abs},8}$	$f^{\text{abs},8}$	$\omega_{S}^{\text{abs},11}$	$f^{\text{abs},11}$					$\omega_{S}^{\text{abs},8}$	$f^{\text{abs},8}$	$\omega_{S}^{\text{abs},11}$	$f^{\text{abs},11}$		
B3LYP	3.29	1.255	4.17	0.764	4.36	0.753	2.35	0.916	3.25	1.473	4.16	0.931	4.32	0.704	2.36	1.300
			$\omega_{S}^{\text{abs},8}$	$f^{\text{abs},8}$	$\omega_{S}^{\text{abs},11}$	$f^{\text{abs},11}$					$\omega_{S}^{\text{abs},8}$	$f^{\text{abs},8}$	$\omega_{S}^{\text{abs},11}$	$f^{\text{abs},11}$		
PBE0	3.41	1.346	4.36	1.160	4.56	0.415	2.27	0.909	3.37	1.575	4.33	1.431	4.53	0.299	2.28	1.286
									$\omega_{1\mathrm{st}}^{\text{abs}}$		$\omega_{2\mathrm{nd}}^{\text{abs}}$					
Expt									3.44		4.35					

**Table 13:** The same as Table 11, but for B2PPQ. Experimental values measured in chloroform are obtained from Ref. [1].

	gas	gas	gas	gas	gas	gas	gas	gas	sol	sol	sol	sol	sol	sol	sol	sol
	$\omega_{S}^{\text{abs},1}$	$f^{\text{abs},1}$	$\omega_{S}^{\text{abs},10}$	$f^{\text{abs},10}$	$\omega_{S}^{\text{abs},11}$	$f^{\text{abs},11}$	$\omega_{T}^{\text{abs}}$	$\mu_{g}$	$\omega_{S}^{\text{abs},1}$	$f^{\text{abs},1}$	$\omega_{S}^{\text{abs},9}$	$f^{\text{abs},9}$	$\omega_{S}^{\text{abs},12}$	$f^{\text{abs},12}$	$\omega_{T}^{\text{abs}}$	$\mu_{\text{sol}}$
LSDA	2.48	1.168	3.04	0.235	3.19	0.306	2.14	0.708	2.44	1.403	3.03	0.404	3.20	0.277	2.14	1.025
			$\omega_{S}^{\text{abs},5}$	$f^{\text{abs},5}$	$\omega_{S}^{\text{abs},9}$	$f^{\text{abs},9}$					$\omega_{S}^{\text{abs},5}$	$f^{\text{abs},5}$	$\omega_{S}^{\text{abs},15}$	$f^{\text{abs},15}$		
TPSS	2.62	1.135	3.19	0.366	3.35	0.261	2.11	0.828	2.59	1.332	3.17	0.590	3.62	0.193	2.12	1.227
			$\omega_{S}^{\text{abs},7}$	$f^{\text{abs},7}$	$\omega_{S}^{\text{abs},13}$	$f^{\text{abs},13}$					$\omega_{S}^{\text{abs},7}$	$f^{\text{abs},7}$	$\omega_{S}^{\text{abs},13}$	$f^{\text{abs},13}$		
TPSSh	2.95	1.446	3.73	0.336	4.05	0.607	2.18	0.847	2.91	1.695	3.75	0.442	4.01	0.863	2.19	1.244
			$\omega_{S}^{\text{abs},8}$	$f^{\text{abs},8}$	$\omega_{S}^{\text{abs},13}$	$f^{\text{abs},13}$					$\omega_{S}^{\text{abs},6}$	$f^{\text{abs},6}$	$\omega_{S}^{\text{abs},13}$	$f^{\text{abs},13}$		
B3LYP	3.18	1.690	4.01	0.692	4.32	0.876	2.31	0.908	3.14	1.952	3.98	0.851	4.29	0.885	2.32	1.308
			$\omega_{S}^{\text{abs},8}$	$f^{\text{abs},8}$	$\omega_{S}^{\text{abs},13}$	$f^{\text{abs},13}$					$\omega_{S}^{\text{abs},8}$	$f^{\text{abs},8}$	$\omega_{S}^{\text{abs},13}$	$f^{\text{abs},13}$		
PBE0	3.31	1.812	4.18	1.155	4.52	0.808	2.23	0.889	3.26	2.082	4.18	0.753	4.49	0.688	2.25	1.287
									$\omega_{1\mathrm{st}}^{\text{abs}}$		$\omega_{2\mathrm{nd}}^{\text{abs}}$					
Expt									3.39		4.22					

**Table 14:** The same as Table 11, but for BDBPQ. Experimental values measured in chloroform are obtained from Ref. [1].

	gas	gas	gas	gas	gas	gas	gas	gas	sol	sol	sol	sol	sol	sol	sol	sol
	$\omega_{S}^{\text{abs},1}$	$f^{\text{abs},1}$	$\omega_{S}^{\text{abs},10}$	$f^{\text{abs},10}$	$\omega_{S}^{\text{abs},11}$	$f^{\text{abs},11}$	$\omega_{T}^{\text{abs}}$	$\mu_{g}$	$\omega_{S}^{\text{abs},1}$	$f^{\text{abs},1}$	$\omega_{S}^{\text{abs},9}$	$f^{\text{abs},9}$	$\omega_{S}^{\text{abs},12}$	$f^{\text{abs},12}$	$\omega_{T}^{\text{abs}}$	$\mu_{\text{sol}}$
LSDA	2.63	0.683	2.70	0.173	3.26	0.149	2.25	0.772	2.61	0.896	2.70	0.140	3.31	0.131	2.26	1.092
			$\omega_{S}^{\text{abs},12}$	$f^{\text{abs},12}$	$\omega_{S}^{\text{abs},14}$	$f^{\text{abs},14}$					$\omega_{S}^{\text{abs},10}$	$f^{\text{abs},10}$	$\omega_{S}^{\text{abs},14}$	$f^{\text{abs},14}$		
TPSS	2.76	0.797	3.36	0.120	3.46	0.246	2.18	0.891	2.74	0.957	3.37	0.143	3.44	0.266	2.19	1.231
			$\omega_{S}^{\text{abs},13}$	$f^{\text{abs},13}$	$\omega_{S}^{\text{abs},15}$	$f^{\text{abs},15}$					$\omega_{S}^{\text{abs},6}$	$f^{\text{abs},6}$	$\omega_{S}^{\text{abs},13}$	$f^{\text{abs},13}$		
TPSSh	3.08	1.069	3.85	0.217	3.91	0.138	2.22	0.922	3.05	1.236	3.57	0.131	3.86	0.284	2.23	1.262
			$\omega_{S}^{\text{abs},12}$	$f^{\text{abs},12}$	$\omega_{S}^{\text{abs},14}$	$f^{\text{abs},14}$					$\omega_{S}^{\text{abs},5}$	$f^{\text{abs},5}$	$\omega_{S}^{\text{abs},12}$	$f^{\text{abs},12}$		
B3LYP	3.31	1.227	4.14	0.446	4.27	0.153	2.36	1.014	3.28	1.400	3.83	0.171	4.13	0.436	2.37	1.361
			$\omega_{S}^{\text{abs},10}$	$f^{\text{abs},10}$	$\omega_{S}^{\text{abs},13}$	$f^{\text{abs},13}$					$\omega_{S}^{\text{abs},10}$	$f^{\text{abs},10}$	$\omega_{S}^{\text{abs},13}$	$f^{\text{abs},13}$		
PBE0	3.42	1.321	4.31	0.644	4.45	0.270	2.27	0.982	3.39	1.501	4.29	0.842	4.44	0.407	2.28	1.335
									$\omega_{1\mathrm{st}}^{\text{abs}}$		$\omega_{2\mathrm{nd}}^{\text{abs}}$					
Expt									3.45		4.40					

**Table 15:** The same as Table 11, but for BPYPQ. The basis set 6-31G(d) is used. Experimental values measured in chloroform are obtained from Ref. [108].

	gas	gas	gas	gas	gas	gas	gas	gas	sol	sol	sol	sol	sol	sol	sol	sol
	$\omega_{S}^{\text{abs},1}$	$f^{\text{abs},1}$	$\omega_{S}^{\text{abs},4}$	$f^{\text{abs},4}$	$\omega_{S}^{\text{abs},14}$	$f^{\text{abs},14}$	$\omega_{T}^{\text{abs}}$	$\mu_{g}$	$\omega_{S}^{\text{abs},1}$	$f^{\text{abs},1}$	$\omega_{S}^{\text{abs},4}$	$f^{\text{abs},4}$	$\omega_{S}^{\text{abs},12}$	$f^{\text{abs},12}$	$\omega_{T}^{\text{abs}}$	$\mu_{\text{sol}}$
LSDA	2.10	0.558	2.48	0.358	3.06	0.286	1.90	1.031	2.08	0.714	2.47	0.499	3.03	0.384	1.90	1.645
			$\omega_{S}^{\text{abs},3}$	$f^{\text{abs},3}$	$\omega_{S}^{\text{abs},12}$	$f^{\text{abs},12}$				$\omega_{S}^{\text{abs},5}$	$f^{\text{abs},5}$	$\omega_{S}^{\text{abs},12}$	$f^{\text{abs},12}$			
TPSS	2.21	0.473	2.58	0.392	3.17	0.334	1.85	1.112	2.19	0.610	2.56	0.536	3.15	0.400	1.86	1.761
			$\omega_{S}^{\text{abs},5}$	$f^{\text{abs},5}$	$\omega_{S}^{\text{abs},11}$	$f^{\text{abs},11}$				$\omega_{S}^{\text{abs},5}$	$f^{\text{abs},5}$	$\omega_{S}^{\text{abs},10}$	$f^{\text{abs},10}$			
TPSSh	2.61	0.852	3.16	0.506	3.48	0.271	1.88	1.138	2.58	1.081	3.14	0.503	3.45	0.314	1.89	1.784
			$\omega_{S}^{\text{abs},5}$	$f^{\text{abs},5}$	$\omega_{S}^{\text{abs},9}$	$f^{\text{abs},9}$					$\omega_{S}^{\text{abs},5}$	$f^{\text{abs},5}$	$\omega_{S}^{\text{abs},7}$	$f^{\text{abs},7}$		
B3LYP	2.89	1.275	3.44	0.501	3.64	0.092	1.98	1.122	2.85	1.998	3.42	0.513	3.61	0.122	1.98	1.744
			$\omega_{S}^{\text{abs},5}$	$f^{\text{abs},5}$	$\omega_{S}^{\text{abs},9}$	$f^{\text{abs},9}$					$\omega_{S}^{\text{abs},5}$	$f^{\text{abs},5}$	$\omega_{S}^{\text{abs},14}$	$f^{\text{abs},14}$		
PBE0	3.04	1.516	3.62	0.448	3.79	0.071	1.88	1.190	3.00	1.817	3.60	0.479	4.09	0.096	1.89	1.839
									$\omega_{1\mathrm{st}}^{\text{abs}}$		$\omega_{2\mathrm{nd}}^{\text{abs}}$		$\omega_{3\mathrm{rd}}^{\text{abs}}$			
Expt									3.26		3.60		4.34			

**Table 16:** The same as Table 11, but for B3PPQ. The basis set 6-31G(d) is used. Experimental values measured in chloroform are obtained from Ref. [108].

	gas	gas	gas	gas	gas	gas	gas	gas	sol	sol	sol	sol	sol	sol	sol	sol
	$\omega_{S}^{\text{abs},1}$	$f^{\text{abs},1}$	$\omega_{S}^{\text{abs},4}$	$f^{\text{abs},5}$	$\omega_{S}^{\text{abs},13}$	$f^{\text{abs},13}$	$\omega_{T}^{\text{abs}}$	$\mu_{g}$	$\omega_{S}^{\text{abs},1}$	$f^{\text{abs},1}$	$\omega_{S}^{\text{abs},5}$	$f^{\text{abs},5}$	$\omega_{S}^{\text{abs},13}$	$f^{\text{abs},13}$	$\omega_{T}^{\text{abs}}$	$\mu_{\text{sol}}$
LSDA	2.34	1.235	2.78	0.598	3.27	0.575	2.09	1.137	2.31	1.421	2.75	0.721	3.26	0.593	2.09	1.457
			$\omega_{S}^{\text{abs},4}$	$f^{\text{abs},4}$	$\omega_{S}^{\text{abs},13}$	$f^{\text{abs},13}$				$\omega_{S}^{\text{abs},4}$	$f^{\text{abs},4}$	$\omega_{S}^{\text{abs},14}$	$f^{\text{abs},14}$		
TPSS	2.49	1.209	2.93	0.425	3.42	0.503	2.079	1.234	2.46	1.378	2.89	0.430	3.42	0.391	2.09	1.602
			$\omega_{S}^{\text{abs},5}$	$f^{\text{abs},5}$	$\omega_{S}^{\text{abs},16}$	$f^{\text{abs},16}$				$\omega_{S}^{\text{abs},10}$	$f^{\text{abs},10}$	$\omega_{S}^{\text{abs},15}$	$f^{\text{abs},15}$		
TPSSh	2.86	1.779	3.42	0.502	3.95	1.047	2.16	1.267	2.83	1.998	3.74	0.772	3.92	0.856	2.18	1.636
			$\omega_{S}^{\text{abs},7}$	$f^{\text{abs},7}$	$\omega_{S}^{\text{abs},12}$	$f^{\text{abs},12}$				$\omega_{S}^{\text{abs},6}$	$f^{\text{abs},6}$	$\omega_{S}^{\text{abs},11}$	$f^{\text{abs},11}$		
B3LYP	3.12	2.197	3.86	0.542	4.09	0.923	2.29	1.265	3.08	2.429	3.81	0.498	4.06	0.642	2.30	1.632
			$\omega_{S}^{\text{abs},6}$	$f^{\text{abs},6}$	$\omega_{S}^{\text{abs},12}$	$f^{\text{abs},12}$				$\omega_{S}^{\text{abs},6}$	$f^{\text{abs},6}$	$\omega_{S}^{\text{abs},12}$	$f^{\text{abs},12}$		
PBE0	3.27	2.373	4.02	0.957	4.30	0.962	2.23	1.306	3.23	2.609	4.02	0.842	4.28	1.099	2.24	1.676
									$\omega_{1\mathrm{st}}^{\text{abs}}$		$\omega_{2\mathrm{nd}}^{\text{abs}}$					
Expt									3.32		4.04					

**Table 17:** TDDFT natural transition orbital analysis for the three excited states with the largest oscillator strengths in B1PPQ in gas phase. Δ*E* is the excitation energy, *f* is the corresponding oscillator strength, and *W* is the weight of the plotted orbital in the respective transition density matrix.

Excited state	Electron	Hole
|1〉 Δ*E* = 3.45 eV *f* = 1.112 *W* = 97.4%	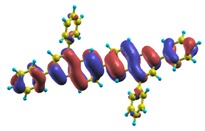	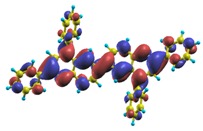
|8〉 Δ*E* = 4.41 eV f = 0.786 *W* = 53.5%	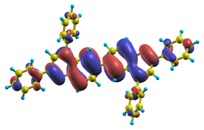	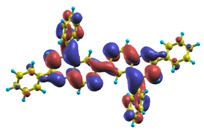
|9〉 Δ*E* = 4.54 eV f = 0.744 *W* = 81.8%	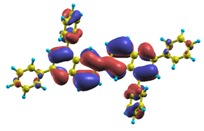	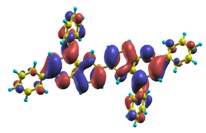

**Table 18:** The same as Table 17, but for BtBPQ.

Excited state	Electron	Hole
|1〉 Δ*E* = 3.41 eV *f* = 1.346 *W* = 97.2%	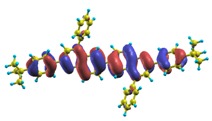	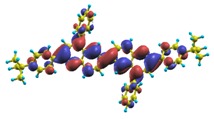
|8〉 Δ*E* = 4.36 eV *f* = 1.160 *W* = 58.7%	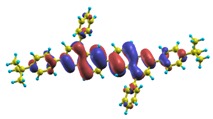	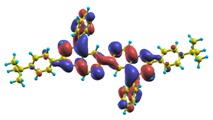
|11〉 Δ*E* = 4.56 eV *f* = 0.415 *W* = 82.5%	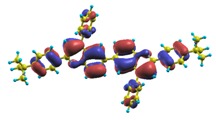	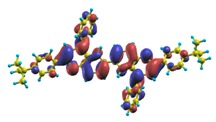

**Table 19:** Excitation energies of singlet-singlet (*S*_0_ − *S*_1_) and singlet-triplet (*S*_0_ − *T*_1_) gaps (in units of eV) of polymers of length of ∼ 10 nm in gas phase calculated using the adiabatic TDDFT methods with the ground-state geometries optimized on the respective density functionals. Basis set 6-31G is used in all calculations. The number in parentheses is the number of rings included in our calculations. 1 hartree = 27.21 eV.

	*S*_0_ − *S*_1_							$S_{0}-T_{1}^{a}$					
Polymer	Expt^*a*^	LSDA	TPSS	TPSSh	B3LYP	PBE0		Expt^*a*^	LSDA	TPSS	TPSSh	B3LYP	PBE0
P3OT(28)	2.8-3.8	0.99	0.99	1.35	1.59	1.76		1.7-2.2	0.90	0.80	0.88	0.96	0.95
PBOPT(32)	2.52	1.49	1.55	1.96	2.26	2.39		1.60	1.37	1.31	1.42	1.57	1.54
MEHPPV(16)	2.48	1.14	1.27	1.66	1.94	2.07		1.30	1.04	1.08	1.18	1.31	1.24
PFO(36)	3.22	2.30	2.45	2.89	3.13	3.30		2.30	2.22	2.23	2.34	2.45	2.43
DHOPPV(16)	2.58	1.14	1.27	1.67	1.95	2.07		1.50	1.04	1.08	1.18	1.32	1.24
PPY(24)	3.4-3.9	1.82	2.10	2.61	2.87	3.03		2.4-2.5	1.82	1.99	2.11	2.23	2.20
CN-MEHPPV(16)	2.72	1.10	1.34	1.84	2.16	2.27		N/A	1.06	1.22	1.34	1.48	1.43
PANi(20)	2.00	2.34	2.53	3.05	3.30	3.44		< 0.9	2.31	2.43	2.63	2.75	2.73

^*a*^From Ref. [120], in which there is a small redshift in gas phase, compared to those in solvent (see discussion in the context). ^*b*^Notation of Ref. [132] is used. Note that all the groups of -(CH_2_)_*n*_CH_3_ in polymers have been replaced with the hydrogen (-H).

**Table 20:** Torsions of the conjugated polymers.

Polymer	Expt	PBE0	Energy
P3OT^*a*^	∼ 24 °	∼ 0 °	redshift
PBOPT	∼ 35 °	∼ 40 °	On experiment
MEHPPV^*b*^	∼ 30 °	∼ 1 °	redshift
PFO^*c*^	∼ 40 °	∼ 38 °	On experiment
DHOPPV	∼ 30 °	∼ 0 °	redshift
PPY^*d*^	≳ 0 °	∼ 0 − 1 °	slightly redshift
CN-MEHPPV	∼ 30 °	∼ 0 °	redshift
PANi	∼ 0 °	∼ 18 − 26 °	too blueshift

^*a*^From Ref. [141,142]. ^*b*^From Ref. [143]. ^*c*^From Ref. [31]. ^*d*^From Ref. [144].

**Table 21:** The same as Table 19, but in benzene solution. The solvent effects are taken into account through PCM (polarizable continuum model) method.

	*S*_0_ − *S*_1_							$S_{0}-T_{1}^{b}$					
Polymer	Expt^*a*^	LSDA	TPSS	TPSSh	B3LYP	PBE0		Expt^*a*^	LSDA	TPSS	TPSSh	B3LYP	PBE0
P3OT(28)	2.8-3.8	0.97	0.97	1.32	1.56	1.73		1.7-2.2	0.89	0.80	0.87	0.95	0.94
PBOPT(32)	2.52							1.60					
MEHPPV(16)	2.48	1.12	1.25	1.64	1.91	2.04		1.30	1.03	1.07	1.18	1.32	1.25
PFO(36)	3.22	2.30	2.45	2.88	3.12	3.29		2.30	2.22	2.24	2.35	2.46	2.43
DHOPPV(16)	2.58	1.12	1.25	1.64	1.92	2.04		1.50	1.03	1.07	1.18	1.32	1.25
PPY(24)	3.4-3.9	2.08	2.16	2.61	2.85	3.01		2.4-2.5	2.02	1.99	2.11	2.23	2.20
CN-MEHPPV(16)	2.72	1.10	1.32	1.80	2.10	2.21		N/A	1.05	1.21	1.34	1.48	1.43
PANi(20)	2.00	2.33	2.53	3.03	3.27	3.41		< 0.9	2.30	2.42	2.62	2.75	2.73

^*a*^From Ref. [120].

^*b*^Notation of Ref. [132] is used. Note that all the groups of -(CH_2_)_*n*_CH_3_ in polymers have been replaced with the hydrogen (-H).
